# Crystal structures of two alanyl­piperidine analogues

**DOI:** 10.1107/S2056989021010392

**Published:** 2021-10-13

**Authors:** Kalina Mambourg, Nikolay Tumanov, Gilles Henon, Steve Lanners, Javier Garcia-Ladona, Johan Wouters

**Affiliations:** a Department of Chemistry, University of Namur, Rue de Bruxelles 61, Namur 5000, Belgium; b Abaxys Therapeutics, Rue du Berceau 91, Villers-la-Ville 1495, Belgium

**Keywords:** crystal structure, UCH-L1 activator, alanyl­piperidine derivatives, polymorph risk assessment

## Abstract

The structure of ethyl 1-[*N*-(4-methyl­phen­yl)-*N*-(methyl­sulfon­yl)alan­yl]piperidine-4-carboxyl­ate, C_19_H_28_N_2_O_5_S, **I**, a compound of inter­est as activator of Ubiquitin C-terminal Hydro­lase-L1 (UCH-L1), was determined by single-crystal X-ray diffraction (SCXRD) analysis. In order to find new activators, a derivative of compound **I**, namely, 1-[*N*-(4-methyl­phen­yl)-*N*-(methyl­sulfon­yl)alan­yl]piperidine-4-carb­oxy­lic acid, C_17_H_24_N_2_O_5_S, **II**, was studied. The synthesis and crystal structure are also reported. Despite being analogues, different crystal packings are observed. Compound **II** bears a carb­oxy­lic group, which favors a strong hydrogen bond. A polymorph risk assessment was carried out to study inter­actions in compound **II**.

## Abstract 2

The crystal structures of ethyl 1-[*N*-(4-methyl­phen­yl)-*N*-(methyl­sulfon­yl)alan­yl]piperidine-4-carboxyl­ate and 1-[*N*-(4-methyl­phen­yl)-*N*-(methyl­sulfon­yl)alan­yl]piperidine-4-carb­oxy­lic acid, two analogues studied as potentiators of Ubiquitin C-terminal hydro­lase-L1 (UCH-L1), have been determined. Despite being analogues, different crystal packings are observed. A polymorph risk assessment was carried out to study inter­actions in the second compound.

## Chemical context

Ubiquitin C-terminal Hydro­lase-L1 is a deubiquitinase that represents 2% of the neuronal soluble proteins in the brain and is involved in the neuropathogenesis of neurodegenerative diseases. Studies have shown that several mutations have an impact on the hydro­lase activity of UCH-L1 (Leroy *et al.*, 1998 ▸; Maraganore *et al.*, 1999 ▸) and that its down-regulation is associated with idiopathic Parkinson’s disease (Choi *et al.*, 2004 ▸). Finding potentiators of UCH-L1 could be a therapeutic pathway for these diseases (Mitsui *et al.*, 2010 ▸). Ethyl 1-[*N*-(methyl­sulfon­yl)-*N*-(*p*-tol­yl)-alan­yl]piperidine-4-carboxyl­ate was discovered through *in silico* drug screening as an activator of UCH-L1, with a hydro­lase activity up to 111% at 63 µ*M* (Mitsui *et al.*, 2010 ▸). We studied the only known activator in the literature, compound **I**. Derivatives of compound **I** were then investigated as potential activators and compound **II** was obtained after a saponification. Compound **II** bears a carb­oxy­lic acid group, which opens up the possibility for co-crystallization and salification in order to modulate the physicochemical properties, such as the solubility. We report the crystal structures of these two compounds as well as a survey of the inter­actions observed in compound **II**. 
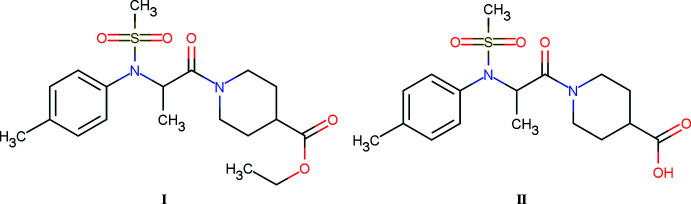


## Structural commentary

**Figure 1:**
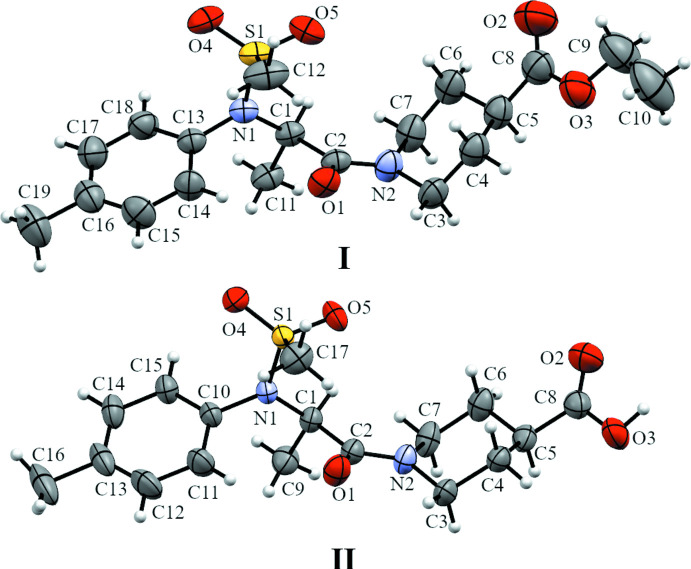
The asymmetric units of compounds **I** and **II**, with displacement ellipsoids drawn at the 50% probability level.

Both compounds crystallize as colorless plate-like crystals but in different space groups. Compound **I** crystallizes in the triclinic *P* 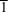 space groups and compound **II** in the monoclinic *P*2_1_/*n* space group. The asymmetric units are shown in Fig. 1 ▸. Both compounds crystallize as a racemic mixture and have one mol­ecule in the asymmetric unit in a similar conformation. The torsion angle N1—C1—C2—N2 is 156.2 (1) and −153.5 (1)° for **I** and **II** respectively. The only slight difference between the two compounds is the geometry of N2. In compound **I**, the distance between N2 and the plane formed by C2, C3 and C7 is 0.114 (2) Å whereas in compound **II** this distance is 0.014 (2) Å. A more planar arrangement of N2 in compound **II** is noticed, probably caused by the crystal packing. Single crystals represent the bulk samples as the powder patterns calculated from SCXRD data are similar to the experimental ones.

## Supra­molecular features

**Figure 2:**
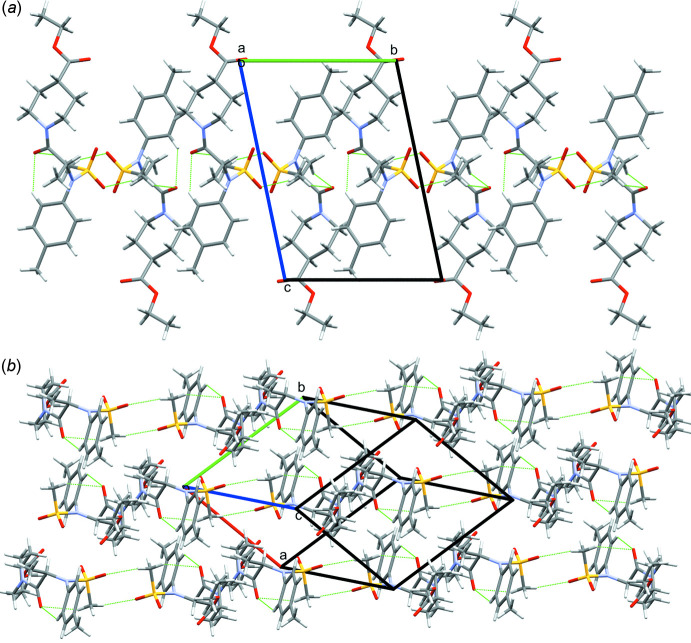
Crystal packing of **I** with hydrogen bonds highlighted in green (*a*) showing one layer of mol­ecules, viewed down the *a* axis and (*b*) showing adjacent layers of mol­ecules.

As compound **I** does not have any strong hydrogen-bond acceptors, only weak hydrogen bonds are observed in the crystal structure (see Table 1 ▸). The amide oxygen atom O1 participates in the formation of two intra­molecular hydrogen bonds [ 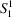 (7) motifs; Etter *et al.*, 1990 ▸]. The oxygen atom O4 is inter-connected with atom H12*C* of the sulfonyl methyl of an adjacent mol­ecule [*d*(H⋯O) 2.44 Å; Table 1 ▸], forming an 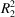 (8) hydrogen bond motif along the *a-*axis direction (Fig. 2 ▸). As compound **I** bears a tolyl moiety, π–π inter­actions were expected but were not observed in this crystal packing.

**Figure 3:**
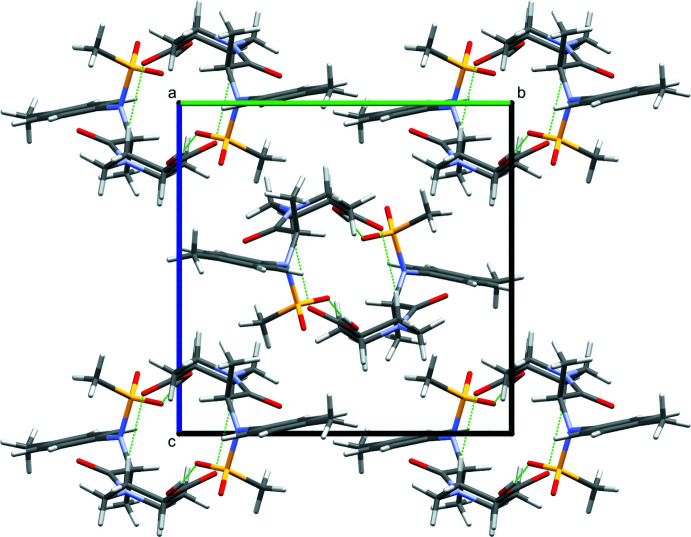
Crystal packing of **II** showing the tubular arrangement viewed down the *a* axis. Hydrogen bonds are highlighted in green.

Compound **II** bearing a carb­oxy­lic moiety instead of an ester has an impact on the hydrogen bonds and thus on the crystal packing. In compound **II**, a tubular arrangement (Fig. 3 ▸) can be observed, which is different from that of compound **I**. In compound **II**, a hydrogen-bonded ring with an 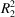 (24) motif is formed by a strong hydrogen bond between H3 of the carb­oxy­lic acid group and O5 from an adjacent mol­ecule [*d*(H⋯O) 1.88 (3) Å; Table 2 ▸]. In addition, two intra­molecular [ 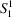 (7) motifs] and one inter­molecular [ 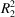 (10) motif] weak hydrogen bonds are detected. As in compound **I**, no π–π inter­actions are noticed in the crystal structure. A dimer synthon is observed in the crystal packing in both cases, but for compound **I** it is ensured by weak hydrogen bonds in contrast to compound **II** where the dimer is based on strong hydrogen bonds.

## Database survey

Searches of the Cambridge Structural Database (CSD, version 5.42, update September 2021; Groom *et al.* 2016 ▸) were carried out with the exact structures of compounds **I** and **II** and with substructures containing the significant fragments (alanyl­piperidine with and without the sulfonyl methyl and tolyl group). No comparable structures came out of this survey.

**Figure 4:**
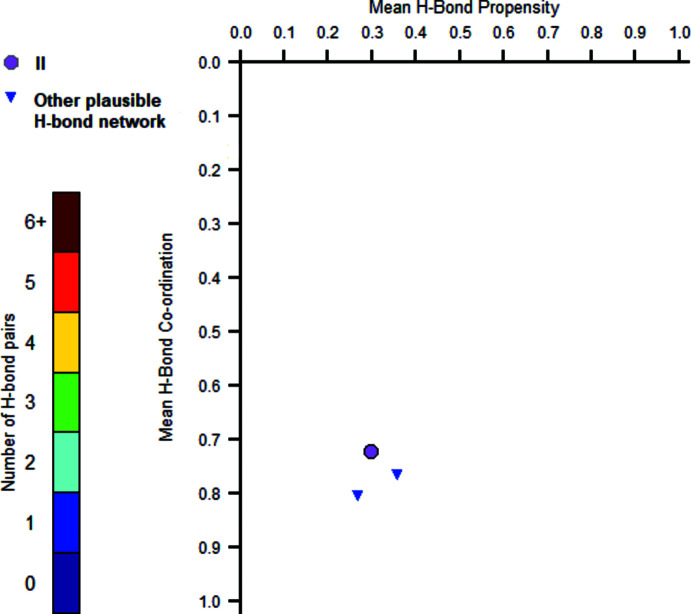
Hydrogen-bond propensity chart for compound **II**.

A polymorph risk assessment based on the hydrogen bonds in the CSD was carried out. This statistical analysis allows us to estimate which atoms are the donors and the acceptors for hydrogen bonds in the crystal structure (Chemburkar *et al.*, 2000 ▸; Galek *et al.*, 2007 ▸). This qu­anti­fies the probability of hydrogen-bond formation and thus the different probable polymorphs that can arise from a specific compound. The results are summarized in Table 3 ▸. A hydrogen-bonding inter­action between two carb­oxy­lic groups is predicted with the highest probability. We did not observe the carb­oxy­lic dimer but rather this group inter­acting with one oxygen of the sulfonyl methyl. The analysis also predicts other plausible hydrogen-bonded networks (Fig. 4 ▸), one that is statistically slightly more likely to be formed than the current one. This suggests that another potential polymorph could be obtained. Thus, we undertook a polymorph screening by several crystallization experiments of compound **II**. The recrystallization solvents that we tested were cyclo­hexane, toluene, ethyl acetate, chloro­form, di­chloro­methane, acetone, aceto­nitrile, 2-propanol, ethanol and methanol. They all lead to the same polymorph.

## Synthesis and crystallization

**Compound I:** This was purchased from Evotech (Hamburg, Germany). The product was crystallized by slow evaporation from non-anhydrous ethyl acetate, which provided colorless plate-like crystals suitable for SCXRD. M.p. 442.2 K

**Compound II:** In a round-bottom flask, compound **I** (405.1 mg, 1.02 mmol, 1.0 eq) dissolved in 8 mL of THF was added to a solution of LiOH (81.9 mg, 3.40 mmol, 3.4 eq) dissolved in 5 mL of water. The mixture was stirred at room temperature for 8 h. The resulting mixture was washed with ether. The aqueous phase was then acidified with HCl 37% to a pH of 2 and extracted with di­chloro­methane. The combined organic phases were dried over anhydrous Na_2_SO_4_ and concentrated under vacuum to yield a white solid (351.0 mg, 93%). The product was crystallized by slow evaporation from methanol, which provided colorless plate-like crystals suitable for SCXRD. ^1^H NMR (DMSO): 12.32 (*s*, 1H, carb­oxy­lic acid), 7.39 (*d*, 2H, CH_arom_), 7.20 (*d*, 2H, CH_arom_), 5.20 (*s*, 1H, CH_α_), 4.03–3.15 (*m*, 4H, CH_pip_), 2.96 (*s*, 3H, CH_SO2Me_), 2.79 (*m*, 1H, CH_pip_), 2.31 (*s*, 3H, CH_PheMe_), 1.83–1.36 (*m*, 4H, CH_pip_), 1.03 (*d*, 3H, CH_αMe_) ^13^C NMR (DMSO): 169.1, 168.7, 138.2, 133.5, 132.0, 129.4, 53.3, 44.5, 41.3, 28.5, 20.7, 16.8. M.p. 496.2 K

## Refinement

Crystal data, data collection and structure refinement details are summarized in Table 4 ▸. All H atoms, except one of the -OH group in **II**, were refined using a riding model, with C—H = 0.93 (aromatic), 0.96 (meth­yl) or 0.98 Å (tertiary carbon). Coordinates of the hydrogen atom of the -OH group were refined. The isotropic atomic displacement parameters of the H atoms were set at 1.5*U*_eq_ of the parent atom for the methyl and alcohol groups, and at 1.2*U*_eq_ otherwise.

## Supplementary Material

Crystal structure: contains datablock(s) I, II. DOI: 10.1107/S2056989021010392/vm2254sup1.cif

Supporting information file. DOI: 10.1107/S2056989021010392/vm2254Isup4.mol

Structure factors: contains datablock(s) I. DOI: 10.1107/S2056989021010392/vm2254Isup6.hkl

Supporting information file. DOI: 10.1107/S2056989021010392/vm2254IIsup5.mol

Structure factors: contains datablock(s) II. DOI: 10.1107/S2056989021010392/vm2254IIsup7.hkl

Supporting information file. DOI: 10.1107/S2056989021010392/vm2254Isup6.cml

Supporting information file. DOI: 10.1107/S2056989021010392/vm2254IIsup7.cml

CCDC references: 2114340, 2114339

Additional supporting information:  crystallographic information; 3D view; checkCIF report

## Figures and Tables

**Table 1:** Hydrogen-bond geometry (Å, °) for compound **I** [Chem scheme1]

*D*—H⋯*A*	*D*—H	H⋯*A*	*D*⋯*A*	*D*—H⋯*A*
C12—H12*B*⋯O1	0.96	2.50	3.210 (2)	130
C12—H12*C*⋯O4^i^	0.96	2.44	3.376 (2)	164
C14—H14⋯O1	0.93	2.48	3.177 (2)	132

Symmetry code: (i) -x, -y, -z+1.

**Table 2:** Hydrogen-bond geometry (Å, °) for compound **II** [Chem scheme1]

*D*—H⋯*A*	*D*—H	H⋯*A*	*D*⋯*A*	*D*—H⋯*A*
O3—H3⋯O5^i^	0.90 (3)	1.88 (3)	2.7463 (15)	161 (2)
C17—H17*A*⋯O1	0.96	2.48	3.144 (2)	127
C4—H4*B*⋯O2^i^	0.97	2.52	3.471 (2)	167
C11—H11⋯O1	0.93	2.56	3.2558 (19)	132

Symmetry code: (i) -x+2, -y+1, -z+1.

**Table 3:** Hydrogen-bond propensity calculation for compound **II**

Donor	Acceptor	Propensity
O3	O2	0.36
O3	O4	0.30
O3	O5	0.30

**Table 4:** Experimental details

	**I**	**II**
Crystal data		
Chemical formula	C_19_H_28_N_2_O_5_S	C_17_H_24_N_2_O_5_S
*M*_r_	396.49	368.44
Crystal system, space group	Triclinic, *P*\overline{1}	Monoclinic, *P*2_1_/*n*
Temperature (K)	295	295
*a*, *b*, *c* (Å)	8.5368 (6), 9.6594 (6), 13.5173 (12)	12.1013 (2), 12.3092 (2), 12.4348 (3)
α, β, γ (°)	75.947 (6), 79.302 (6), 74.554 (5)	90, 100.546 (2), 90
*V* (Å^3^)	1033.47 (14)	1820.97 (6)
*Z*	2	4
Radiation type	Mo *K*α	Mo *K*α
μ (mm^−1^)	0.19	0.21
Crystal size (mm)	0.79 × 0.18 × 0.05	0.77 × 0.18 × 0.11

Data collection		
Diffractometer	Oxford Diffraction Xcalibur, Gemini Ultra R	Oxford Diffraction Xcalibur, Gemini Ultra R
Absorption correction	Analytical [*CrysAlis PRO* (Rigaku OD, 2018 ▸), based on expressions derived by Clark & Reid (1995 ▸)]	Analytical [*CrysAlis PRO* (Rigaku OD, 2018 ▸), based on expressions derived by Clark & Reid (1995 ▸)]
*T*_min_, *T*_max_	0.923, 0.991	0.882, 0.980
No. of measured, independent and observed [*I* > 2σ(*I*)] reflections	13200, 6870, 4304	29518, 6284, 4779
*R*_int_	0.026	0.026
(sin θ/λ)_max_ (Å^−1^)	0.762	0.761

Refinement		
*R*[*F* ^2^ > 2σ(*F* ^2^)], *wR*(*F* ^2^), *S*	0.054, 0.158, 1.02	0.043, 0.126, 1.02
No. of reflections	6870	6284
No. of parameters	248	232
H-atom treatment	H-atom parameters constrained	H atoms treated by a mixture of independent and constrained refinement
Δρ_max_, Δρ_min_ (e Å^−3^)	0.34, −0.38	0.29, −0.29

Computer programs: *CrysAlis PRO* (Rigaku OD, 2018 ▸), *SHELXT2014* (Sheldrick, 2015*a* ▸), *SHELXL2016* (Sheldrick, 2015*b* ▸), *Mercury* (Macrae *et al.*, 2020 ▸) and *publCIF* (Westrip, 2010 ▸).

## supplementary crystallographic information

### Ethyl 1-[N-(4-methylphenyl)-N-(methylsulfonyl)alanyl]piperidine-4-carboxylate (I) Crystal data

**Table:**

C_19_H_28_N_2_O_5_S	*Z* = 2
*M**_r_* = 396.49	*F*(000) = 424
Triclinic, *P*1	*D*_x_ = 1.274 Mg m^−^^3^
*a* = 8.5368 (6) Å	Mo *K*α radiation, λ = 0.71073 Å
*b* = 9.6594 (6) Å	Cell parameters from 3246 reflections
*c* = 13.5173 (12) Å	θ = 2.4–30.5°
α = 75.947 (6)°	µ = 0.19 mm^−^^1^
β = 79.302 (6)°	*T* = 295 K
γ = 74.554 (5)°	Plate, colorless
*V* = 1033.47 (14) Å^3^	0.79 × 0.18 × 0.05 mm

### Ethyl 1-[N-(4-methylphenyl)-N-(methylsulfonyl)alanyl]piperidine-4-carboxylate (I) Data collection

**Table:**

Oxford Diffraction Xcalibur, Gemini Ultra R diffractometer	6870 independent reflections
Radiation source: fine-focus sealed X-ray tube	4304 reflections with *I* > 2σ(*I*)
Graphite monochromator	*R*_int_ = 0.026
Detector resolution: 10.3712 pixels mm^-1^	θ_max_ = 32.8°, θ_min_ = 2.2°
ω scans	*h* = −12→10
Absorption correction: analytical [CrysAlisPro (Rigaku OD, 2018), based on expressions derived by Clark & Reid (1995)]	*k* = −13→14
*T*_min_ = 0.923, *T*_max_ = 0.991	*l* = −20→19
13200 measured reflections	

### Ethyl 1-[N-(4-methylphenyl)-N-(methylsulfonyl)alanyl]piperidine-4-carboxylate (I) Refinement

**Table:**

Refinement on *F*^2^	Primary atom site location: dual
Least-squares matrix: full	Secondary atom site location: dual
*R*[*F*^2^ > 2σ(*F*^2^)] = 0.054	Hydrogen site location: mixed
*wR*(*F*^2^) = 0.158	H-atom parameters constrained
*S* = 1.02	*w* = 1/[σ^2^(*F*_o_^2^) + (0.0633*P*)^2^ + 0.1206*P*] where *P* = (*F*_o_^2^ + 2*F*_c_^2^)/3
6870 reflections	(Δ/σ)_max_ < 0.001
248 parameters	Δρ_max_ = 0.34 e Å^−^^3^
0 restraints	Δρ_min_ = −0.37 e Å^−^^3^

### Ethyl 1-[N-(4-methylphenyl)-N-(methylsulfonyl)alanyl]piperidine-4-carboxylate (I) Special details

**Table:**

Geometry. All esds (except the esd in the dihedral angle between two l.s. planes) are estimated using the full covariance matrix. The cell esds are taken into account individually in the estimation of esds in distances, angles and torsion angles; correlations between esds in cell parameters are only used when they are defined by crystal symmetry. An approximate (isotropic) treatment of cell esds is used for estimating esds involving l.s. planes.

### Ethyl 1-[N-(4-methylphenyl)-N-(methylsulfonyl)alanyl]piperidine-4-carboxylate (I) Fractional atomic coordinates and isotropic or equivalent isotropic displacement parameters (Å^2^)

**Table:**

	*x*	*y*	*z*	*U*_iso_*/*U*_eq_	
S1	0.21006 (5)	0.11226 (4)	0.49970 (3)	0.04597 (14)	
O1	0.20546 (16)	0.42592 (12)	0.58010 (10)	0.0542 (3)	
O2	0.2688 (3)	−0.0438 (2)	1.00683 (16)	0.1150 (8)	
O3	0.1551 (3)	0.16259 (18)	1.06193 (12)	0.0894 (5)	
O4	0.21136 (17)	0.03480 (13)	0.42186 (11)	0.0618 (4)	
O5	0.25664 (18)	0.02943 (13)	0.59697 (11)	0.0624 (4)	
N1	0.33461 (16)	0.22121 (13)	0.45549 (10)	0.0404 (3)	
N2	0.3882 (2)	0.31933 (16)	0.69243 (11)	0.0543 (4)	
C1	0.4352 (2)	0.24222 (16)	0.52607 (12)	0.0407 (3)	
H1	0.484975	0.145601	0.564865	0.049*	
C2	0.3309 (2)	0.33501 (16)	0.60307 (13)	0.0435 (4)	
C3	0.2942 (3)	0.4081 (2)	0.76676 (14)	0.0622 (5)	
H3A	0.212739	0.487753	0.733487	0.075*	
H3B	0.367076	0.450568	0.791143	0.075*	
C4	0.2099 (3)	0.3155 (2)	0.85723 (15)	0.0598 (5)	
H4A	0.126979	0.283509	0.834039	0.072*	
H4B	0.155721	0.374364	0.908049	0.072*	
C5	0.3330 (3)	0.1809 (2)	0.90679 (14)	0.0595 (5)	
H5	0.409068	0.216659	0.934903	0.071*	
C6	0.4334 (3)	0.0960 (2)	0.82547 (15)	0.0631 (5)	
H6A	0.517049	0.016237	0.856466	0.076*	
H6B	0.362540	0.054116	0.798864	0.076*	
C7	0.5139 (3)	0.1963 (2)	0.73772 (15)	0.0617 (5)	
H7A	0.590425	0.233317	0.763271	0.074*	
H7B	0.574515	0.141684	0.685678	0.074*	
C8	0.2494 (3)	0.0852 (2)	0.99538 (17)	0.0714 (6)	
C9	0.0650 (4)	0.0879 (3)	1.1529 (2)	0.1069 (10)	
H9A	0.004626	0.029494	1.132728	0.128*	
H9B	0.140898	0.023064	1.198758	0.128*	
C10	−0.0456 (5)	0.1958 (4)	1.2040 (2)	0.1357 (14)	
H10A	0.012297	0.263307	1.213528	0.204*	
H10B	−0.091589	0.148539	1.269779	0.204*	
H10C	−0.131975	0.248210	1.163132	0.204*	
C11	0.5719 (2)	0.31422 (19)	0.46630 (14)	0.0503 (4)	
H11A	0.636931	0.256036	0.418334	0.075*	
H11B	0.639730	0.321567	0.513158	0.075*	
H11C	0.525388	0.410564	0.429625	0.075*	
C12	0.0118 (2)	0.2217 (2)	0.5200 (2)	0.0700 (6)	
H12A	−0.017746	0.283965	0.455966	0.105*	
H12B	0.008836	0.280954	0.568219	0.105*	
H12C	−0.064229	0.159986	0.546811	0.105*	
C13	0.3206 (2)	0.31143 (16)	0.35381 (13)	0.0431 (4)	
C14	0.2231 (2)	0.45197 (18)	0.33966 (14)	0.0543 (4)	
H14	0.166814	0.491483	0.396039	0.065*	
C15	0.2097 (3)	0.5336 (2)	0.24101 (16)	0.0641 (5)	
H15	0.144679	0.628604	0.232086	0.077*	
C16	0.2899 (3)	0.4784 (2)	0.15543 (15)	0.0617 (5)	
C17	0.3898 (3)	0.3385 (2)	0.17173 (16)	0.0675 (6)	
H17	0.447572	0.299605	0.115395	0.081*	
C18	0.4060 (3)	0.25519 (19)	0.26953 (14)	0.0569 (5)	
H18	0.474265	0.161520	0.278607	0.068*	
C19	0.2674 (4)	0.5688 (3)	0.04839 (17)	0.0908 (8)	
H19A	0.162225	0.569619	0.032294	0.136*	
H19B	0.351973	0.526722	−0.000565	0.136*	
H19C	0.273737	0.667492	0.045582	0.136*	

### Ethyl 1-[N-(4-methylphenyl)-N-(methylsulfonyl)alanyl]piperidine-4-carboxylate (I) Atomic displacement parameters (Å^2^)

**Table:**

	*U*^11^	*U*^22^	*U*^33^	*U*^12^	*U*^13^	*U*^23^
S1	0.0381 (2)	0.0362 (2)	0.0636 (3)	−0.00995 (16)	−0.00197 (19)	−0.01207 (17)
O1	0.0444 (7)	0.0491 (6)	0.0645 (8)	0.0048 (5)	−0.0079 (6)	−0.0194 (5)
O2	0.157 (2)	0.0689 (11)	0.1072 (15)	−0.0226 (12)	0.0015 (14)	−0.0131 (10)
O3	0.1050 (15)	0.0800 (10)	0.0695 (10)	−0.0174 (10)	0.0144 (9)	−0.0133 (8)
O4	0.0598 (9)	0.0532 (7)	0.0829 (9)	−0.0212 (6)	−0.0043 (7)	−0.0280 (6)
O5	0.0629 (9)	0.0520 (7)	0.0669 (8)	−0.0211 (6)	−0.0053 (7)	0.0039 (6)
N1	0.0352 (7)	0.0367 (6)	0.0492 (7)	−0.0082 (5)	−0.0054 (6)	−0.0089 (5)
N2	0.0541 (10)	0.0534 (8)	0.0517 (8)	0.0017 (7)	−0.0076 (7)	−0.0191 (6)
C1	0.0333 (8)	0.0376 (7)	0.0507 (9)	−0.0047 (6)	−0.0051 (7)	−0.0119 (6)
C2	0.0389 (9)	0.0382 (7)	0.0527 (9)	−0.0075 (6)	−0.0036 (7)	−0.0114 (6)
C3	0.0761 (15)	0.0538 (10)	0.0557 (11)	−0.0053 (10)	−0.0049 (10)	−0.0226 (8)
C4	0.0598 (13)	0.0600 (11)	0.0568 (11)	−0.0007 (9)	−0.0072 (9)	−0.0214 (9)
C5	0.0600 (13)	0.0647 (11)	0.0542 (11)	−0.0060 (9)	−0.0145 (9)	−0.0170 (8)
C6	0.0635 (14)	0.0587 (10)	0.0620 (12)	0.0094 (9)	−0.0241 (10)	−0.0167 (9)
C7	0.0490 (12)	0.0749 (12)	0.0598 (11)	0.0038 (9)	−0.0152 (9)	−0.0247 (9)
C8	0.0784 (17)	0.0663 (13)	0.0668 (13)	−0.0071 (11)	−0.0187 (12)	−0.0122 (10)
C9	0.114 (3)	0.0948 (18)	0.0859 (19)	−0.0194 (18)	0.0136 (18)	0.0053 (15)
C10	0.125 (3)	0.143 (3)	0.092 (2)	−0.013 (2)	0.033 (2)	0.009 (2)
C11	0.0363 (9)	0.0539 (9)	0.0644 (11)	−0.0139 (7)	−0.0001 (8)	−0.0200 (8)
C12	0.0332 (10)	0.0571 (11)	0.1202 (19)	−0.0110 (8)	0.0035 (11)	−0.0288 (11)
C13	0.0396 (9)	0.0410 (8)	0.0493 (9)	−0.0094 (7)	−0.0059 (7)	−0.0105 (6)
C14	0.0528 (11)	0.0484 (9)	0.0554 (10)	0.0016 (8)	−0.0105 (9)	−0.0109 (8)
C15	0.0567 (13)	0.0568 (11)	0.0695 (13)	0.0007 (9)	−0.0188 (10)	−0.0030 (9)
C16	0.0607 (13)	0.0713 (12)	0.0540 (11)	−0.0235 (10)	−0.0112 (10)	−0.0024 (9)
C17	0.0791 (16)	0.0693 (12)	0.0534 (11)	−0.0223 (11)	0.0062 (10)	−0.0168 (9)
C18	0.0627 (13)	0.0471 (9)	0.0573 (11)	−0.0109 (8)	0.0035 (9)	−0.0143 (8)
C19	0.092 (2)	0.1060 (19)	0.0624 (14)	−0.0224 (16)	−0.0167 (14)	0.0085 (13)

### Ethyl 1-[N-(4-methylphenyl)-N-(methylsulfonyl)alanyl]piperidine-4-carboxylate (I) Geometric parameters (Å, º)

**Table:**

S1—O5	1.4285 (14)	C7—H7A	0.9700
S1—O4	1.4286 (13)	C7—H7B	0.9700
S1—N1	1.6278 (13)	C9—C10	1.428 (4)
S1—C12	1.7526 (19)	C9—H9A	0.9700
O1—C2	1.227 (2)	C9—H9B	0.9700
O2—C8	1.188 (3)	C10—H10A	0.9600
O3—C8	1.324 (3)	C10—H10B	0.9600
O3—C9	1.462 (3)	C10—H10C	0.9600
N1—C13	1.445 (2)	C11—H11A	0.9600
N1—C1	1.476 (2)	C11—H11B	0.9600
N2—C2	1.346 (2)	C11—H11C	0.9600
N2—C3	1.464 (2)	C12—H12A	0.9600
N2—C7	1.466 (2)	C12—H12B	0.9600
C1—C11	1.519 (2)	C12—H12C	0.9600
C1—C2	1.537 (2)	C13—C14	1.380 (2)
C1—H1	0.9800	C13—C18	1.381 (2)
C3—C4	1.516 (3)	C14—C15	1.382 (3)
C3—H3A	0.9700	C14—H14	0.9300
C3—H3B	0.9700	C15—C16	1.379 (3)
C4—C5	1.535 (3)	C15—H15	0.9300
C4—H4A	0.9700	C16—C17	1.384 (3)
C4—H4B	0.9700	C16—C19	1.514 (3)
C5—C8	1.514 (3)	C17—C18	1.381 (3)
C5—C6	1.526 (3)	C17—H17	0.9300
C5—H5	0.9800	C18—H18	0.9300
C6—C7	1.521 (3)	C19—H19A	0.9600
C6—H6A	0.9700	C19—H19B	0.9600
C6—H6B	0.9700	C19—H19C	0.9600
			
O5—S1—O4	118.43 (8)	O2—C8—O3	123.4 (2)
O5—S1—N1	106.30 (8)	O2—C8—C5	125.1 (2)
O4—S1—N1	108.16 (8)	O3—C8—C5	111.50 (18)
O5—S1—C12	108.50 (11)	C10—C9—O3	108.7 (2)
O4—S1—C12	107.33 (10)	C10—C9—H9A	110.0
N1—S1—C12	107.71 (8)	O3—C9—H9A	110.0
C8—O3—C9	119.1 (2)	C10—C9—H9B	110.0
C13—N1—C1	122.65 (12)	O3—C9—H9B	110.0
C13—N1—S1	117.57 (11)	H9A—C9—H9B	108.3
C1—N1—S1	118.92 (10)	C9—C10—H10A	109.5
C2—N2—C3	119.21 (16)	C9—C10—H10B	109.5
C2—N2—C7	126.42 (15)	H10A—C10—H10B	109.5
C3—N2—C7	112.45 (15)	C9—C10—H10C	109.5
N1—C1—C11	110.73 (13)	H10A—C10—H10C	109.5
N1—C1—C2	111.57 (13)	H10B—C10—H10C	109.5
C11—C1—C2	109.61 (12)	C1—C11—H11A	109.5
N1—C1—H1	108.3	C1—C11—H11B	109.5
C11—C1—H1	108.3	H11A—C11—H11B	109.5
C2—C1—H1	108.3	C1—C11—H11C	109.5
O1—C2—N2	121.94 (15)	H11A—C11—H11C	109.5
O1—C2—C1	120.33 (15)	H11B—C11—H11C	109.5
N2—C2—C1	117.58 (15)	S1—C12—H12A	109.5
N2—C3—C4	110.73 (15)	S1—C12—H12B	109.5
N2—C3—H3A	109.5	H12A—C12—H12B	109.5
C4—C3—H3A	109.5	S1—C12—H12C	109.5
N2—C3—H3B	109.5	H12A—C12—H12C	109.5
C4—C3—H3B	109.5	H12B—C12—H12C	109.5
H3A—C3—H3B	108.1	C14—C13—C18	119.67 (16)
C3—C4—C5	111.24 (18)	C14—C13—N1	121.20 (15)
C3—C4—H4A	109.4	C18—C13—N1	119.13 (14)
C5—C4—H4A	109.4	C13—C14—C15	119.47 (17)
C3—C4—H4B	109.4	C13—C14—H14	120.3
C5—C4—H4B	109.4	C15—C14—H14	120.3
H4A—C4—H4B	108.0	C16—C15—C14	122.01 (18)
C8—C5—C6	112.44 (17)	C16—C15—H15	119.0
C8—C5—C4	111.67 (19)	C14—C15—H15	119.0
C6—C5—C4	110.22 (16)	C15—C16—C17	117.39 (18)
C8—C5—H5	107.4	C15—C16—C19	120.6 (2)
C6—C5—H5	107.4	C17—C16—C19	122.0 (2)
C4—C5—H5	107.4	C18—C17—C16	121.65 (19)
C7—C6—C5	110.66 (16)	C18—C17—H17	119.2
C7—C6—H6A	109.5	C16—C17—H17	119.2
C5—C6—H6A	109.5	C13—C18—C17	119.76 (17)
C7—C6—H6B	109.5	C13—C18—H18	120.1
C5—C6—H6B	109.5	C17—C18—H18	120.1
H6A—C6—H6B	108.1	C16—C19—H19A	109.5
N2—C7—C6	109.70 (17)	C16—C19—H19B	109.5
N2—C7—H7A	109.7	H19A—C19—H19B	109.5
C6—C7—H7A	109.7	C16—C19—H19C	109.5
N2—C7—H7B	109.7	H19A—C19—H19C	109.5
C6—C7—H7B	109.7	H19B—C19—H19C	109.5
H7A—C7—H7B	108.2		
			
O5—S1—N1—C13	176.09 (11)	C2—N2—C7—C6	−103.4 (2)
O4—S1—N1—C13	47.92 (13)	C3—N2—C7—C6	60.5 (2)
C12—S1—N1—C13	−67.78 (14)	C5—C6—C7—N2	−57.7 (2)
O5—S1—N1—C1	−14.24 (14)	C9—O3—C8—O2	−2.2 (4)
O4—S1—N1—C1	−142.40 (12)	C9—O3—C8—C5	−179.8 (2)
C12—S1—N1—C1	101.89 (14)	C6—C5—C8—O2	6.3 (4)
C13—N1—C1—C11	−25.36 (19)	C4—C5—C8—O2	130.8 (3)
S1—N1—C1—C11	165.51 (11)	C6—C5—C8—O3	−176.2 (2)
C13—N1—C1—C2	97.02 (16)	C4—C5—C8—O3	−51.7 (3)
S1—N1—C1—C2	−72.11 (15)	C8—O3—C9—C10	−171.3 (3)
C3—N2—C2—O1	3.0 (3)	C1—N1—C13—C14	−76.6 (2)
C7—N2—C2—O1	165.98 (18)	S1—N1—C13—C14	92.68 (17)
C3—N2—C2—C1	178.66 (15)	C1—N1—C13—C18	103.66 (19)
C7—N2—C2—C1	−18.4 (3)	S1—N1—C13—C18	−87.07 (18)
N1—C1—C2—O1	−28.1 (2)	C18—C13—C14—C15	1.3 (3)
C11—C1—C2—O1	94.89 (19)	N1—C13—C14—C15	−178.45 (17)
N1—C1—C2—N2	156.17 (14)	C13—C14—C15—C16	0.7 (3)
C11—C1—C2—N2	−80.81 (18)	C14—C15—C16—C17	−2.1 (3)
C2—N2—C3—C4	106.1 (2)	C14—C15—C16—C19	177.7 (2)
C7—N2—C3—C4	−59.1 (2)	C15—C16—C17—C18	1.7 (3)
N2—C3—C4—C5	54.5 (2)	C19—C16—C17—C18	−178.1 (2)
C3—C4—C5—C8	−178.38 (17)	C14—C13—C18—C17	−1.7 (3)
C3—C4—C5—C6	−52.6 (2)	N1—C13—C18—C17	178.02 (17)
C8—C5—C6—C7	179.54 (18)	C16—C17—C18—C13	0.2 (3)
C4—C5—C6—C7	54.2 (2)		

### Ethyl 1-[N-(4-methylphenyl)-N-(methylsulfonyl)alanyl]piperidine-4-carboxylate (I) Hydrogen-bond geometry (Å, º)

**Table:**

*D*—H···*A*	*D*—H	H···*A*	*D*···*A*	*D*—H···*A*
C12—H12*B*···O1	0.96	2.50	3.210 (2)	130
C12—H12*C*···O4^i^	0.96	2.44	3.376 (2)	164
C14—H14···O1	0.93	2.48	3.177 (2)	132

Symmetry code: (i) −*x*, −*y*, −*z*+1.

### 1-[N-(4-methylphenyl)-N-(methylsulfonyl)alanyl]piperidine-4-carboxylic acid (II) Crystal data

**Table:**

C_17_H_24_N_2_O_5_S	*F*(000) = 784
*M**_r_* = 368.44	*D*_x_ = 1.344 Mg m^−^^3^
Monoclinic, *P*2_1_/*n*	Mo *K*α radiation, λ = 0.71073 Å
*a* = 12.1013 (2) Å	Cell parameters from 8359 reflections
*b* = 12.3092 (2) Å	θ = 2.7–31.5°
*c* = 12.4348 (3) Å	µ = 0.21 mm^−^^1^
β = 100.546 (2)°	*T* = 295 K
*V* = 1820.97 (6) Å^3^	Plate, colorless
*Z* = 4	0.77 × 0.18 × 0.11 mm

### 1-[N-(4-methylphenyl)-N-(methylsulfonyl)alanyl]piperidine-4-carboxylic acid (II) Data collection

**Table:**

Oxford Diffraction Xcalibur, Gemini Ultra R diffractometer	6284 independent reflections
Radiation source: fine-focus sealed X-ray tube	4779 reflections with *I* > 2σ(*I*)
Graphite monochromator	*R*_int_ = 0.026
Detector resolution: 10.3712 pixels mm^-1^	θ_max_ = 32.7°, θ_min_ = 2.2°
ω scans	*h* = −18→18
Absorption correction: analytical [CrysAlisPro (Rigaku OD, 2018), based on expressions derived by Clark & Reid (1995)]	*k* = −18→17
*T*_min_ = 0.882, *T*_max_ = 0.980	*l* = −17→18
29518 measured reflections	

### 1-[N-(4-methylphenyl)-N-(methylsulfonyl)alanyl]piperidine-4-carboxylic acid (II) Refinement

**Table:**

Refinement on *F*^2^	Primary atom site location: dual
Least-squares matrix: full	Secondary atom site location: dual
*R*[*F*^2^ > 2σ(*F*^2^)] = 0.043	Hydrogen site location: difference Fourier map
*wR*(*F*^2^) = 0.126	H atoms treated by a mixture of independent and constrained refinement
*S* = 1.02	*w* = 1/[σ^2^(*F*_o_^2^) + (0.0593*P*)^2^ + 0.3685*P*] where *P* = (*F*_o_^2^ + 2*F*_c_^2^)/3
6284 reflections	(Δ/σ)_max_ = 0.001
232 parameters	Δρ_max_ = 0.29 e Å^−^^3^
0 restraints	Δρ_min_ = −0.29 e Å^−^^3^

### 1-[N-(4-methylphenyl)-N-(methylsulfonyl)alanyl]piperidine-4-carboxylic acid (II) Special details

**Table:**

Geometry. All esds (except the esd in the dihedral angle between two l.s. planes) are estimated using the full covariance matrix. The cell esds are taken into account individually in the estimation of esds in distances, angles and torsion angles; correlations between esds in cell parameters are only used when they are defined by crystal symmetry. An approximate (isotropic) treatment of cell esds is used for estimating esds involving l.s. planes.

### 1-[N-(4-methylphenyl)-N-(methylsulfonyl)alanyl]piperidine-4-carboxylic acid (II) Fractional atomic coordinates and isotropic or equivalent isotropic displacement parameters (Å^2^)

**Table:**

	*x*	*y*	*z*	*U*_iso_*/*U*_eq_	
S1	0.58667 (2)	0.35673 (2)	0.60942 (2)	0.03513 (9)	
O1	0.68078 (9)	0.19851 (8)	0.41698 (10)	0.0531 (3)	
O2	1.04956 (12)	0.60661 (11)	0.37887 (13)	0.0780 (4)	
O3	1.13713 (10)	0.46783 (11)	0.31848 (13)	0.0732 (4)	
H3	1.195 (2)	0.509 (2)	0.352 (2)	0.110*	
O4	0.50845 (8)	0.37442 (9)	0.68112 (8)	0.0479 (2)	
O5	0.65804 (8)	0.44541 (8)	0.59029 (9)	0.0501 (2)	
N1	0.51748 (8)	0.32221 (9)	0.48974 (8)	0.0344 (2)	
N2	0.73982 (9)	0.33804 (10)	0.32395 (10)	0.0447 (3)	
C1	0.56084 (10)	0.35539 (10)	0.39054 (10)	0.0359 (2)	
H1	0.580679	0.432597	0.397240	0.043*	
C17	0.67424 (14)	0.24889 (13)	0.66359 (13)	0.0555 (4)	
H17A	0.726630	0.233560	0.616194	0.083*	
H17B	0.629402	0.185582	0.669483	0.083*	
H17C	0.714621	0.268508	0.734742	0.083*	
C2	0.66661 (10)	0.29079 (11)	0.37903 (10)	0.0392 (3)	
C3	0.83897 (11)	0.27729 (13)	0.30503 (13)	0.0488 (3)	
H3A	0.832698	0.263103	0.227419	0.059*	
H3B	0.842307	0.208002	0.342640	0.059*	
C4	0.94599 (11)	0.34095 (12)	0.34608 (11)	0.0427 (3)	
H4A	1.009763	0.302094	0.327725	0.051*	
H4B	0.957054	0.347251	0.425085	0.051*	
C5	0.94003 (11)	0.45455 (12)	0.29524 (11)	0.0438 (3)	
H5	0.933185	0.446018	0.215935	0.053*	
C6	0.83536 (12)	0.51356 (12)	0.31663 (13)	0.0510 (3)	
H6A	0.841595	0.526101	0.394520	0.061*	
H6B	0.829392	0.583548	0.280182	0.061*	
C7	0.73079 (12)	0.44690 (14)	0.27497 (12)	0.0510 (4)	
H7A	0.665534	0.483641	0.292748	0.061*	
H7B	0.720557	0.440587	0.196007	0.061*	
C8	1.04549 (13)	0.51874 (13)	0.33621 (12)	0.0489 (3)	
C9	0.47176 (12)	0.34023 (14)	0.28791 (12)	0.0489 (3)	
H9A	0.458064	0.264097	0.275037	0.073*	
H9B	0.497692	0.371751	0.226417	0.073*	
H9C	0.403409	0.375286	0.297628	0.073*	
C10	0.42232 (10)	0.25039 (10)	0.48451 (10)	0.0364 (2)	
C11	0.43049 (14)	0.14088 (12)	0.46161 (15)	0.0541 (4)	
H11	0.498708	0.111490	0.451688	0.065*	
C12	0.33581 (16)	0.07526 (14)	0.45357 (15)	0.0641 (4)	
H12	0.341460	0.001934	0.437384	0.077*	
C13	0.23387 (13)	0.11605 (14)	0.46894 (12)	0.0553 (4)	
C14	0.22767 (12)	0.22528 (14)	0.49295 (13)	0.0519 (4)	
H14	0.159834	0.254119	0.504670	0.062*	
C15	0.32057 (10)	0.29283 (12)	0.49995 (11)	0.0423 (3)	
H15	0.314406	0.366372	0.514958	0.051*	
C16	0.13157 (17)	0.0435 (2)	0.45871 (17)	0.0843 (7)	
H16A	0.085792	0.066468	0.509939	0.126*	
H16B	0.155060	−0.030246	0.473939	0.126*	
H16C	0.088916	0.048332	0.385744	0.126*	

### 1-[N-(4-methylphenyl)-N-(methylsulfonyl)alanyl]piperidine-4-carboxylic acid (II) Atomic displacement parameters (Å^2^)

**Table:**

	*U*^11^	*U*^22^	*U*^33^	*U*^12^	*U*^13^	*U*^23^
S1	0.03062 (14)	0.03481 (15)	0.03976 (16)	−0.00400 (11)	0.00588 (11)	−0.00067 (11)
O1	0.0514 (6)	0.0410 (5)	0.0722 (7)	0.0074 (4)	0.0248 (5)	0.0058 (5)
O2	0.0771 (9)	0.0640 (8)	0.0943 (10)	−0.0098 (7)	0.0193 (7)	−0.0239 (7)
O3	0.0402 (6)	0.0697 (8)	0.1097 (11)	−0.0137 (5)	0.0139 (6)	−0.0303 (7)
O4	0.0448 (5)	0.0571 (6)	0.0436 (5)	−0.0033 (4)	0.0129 (4)	−0.0075 (4)
O5	0.0436 (5)	0.0481 (5)	0.0563 (6)	−0.0190 (4)	0.0033 (4)	0.0010 (4)
N1	0.0293 (4)	0.0375 (5)	0.0373 (5)	−0.0058 (4)	0.0082 (4)	−0.0016 (4)
N2	0.0353 (5)	0.0490 (6)	0.0530 (6)	0.0044 (5)	0.0171 (5)	0.0037 (5)
C1	0.0321 (5)	0.0382 (6)	0.0387 (6)	−0.0001 (5)	0.0102 (4)	0.0020 (5)
C17	0.0548 (8)	0.0583 (9)	0.0515 (8)	0.0155 (7)	0.0051 (7)	0.0093 (7)
C2	0.0345 (6)	0.0428 (6)	0.0414 (6)	0.0005 (5)	0.0103 (5)	−0.0025 (5)
C3	0.0366 (6)	0.0525 (8)	0.0609 (9)	0.0005 (6)	0.0184 (6)	−0.0099 (7)
C4	0.0360 (6)	0.0486 (7)	0.0450 (7)	0.0029 (5)	0.0114 (5)	−0.0049 (5)
C5	0.0388 (6)	0.0551 (8)	0.0387 (6)	−0.0029 (6)	0.0107 (5)	0.0008 (6)
C6	0.0493 (8)	0.0481 (8)	0.0591 (8)	0.0065 (6)	0.0190 (7)	0.0149 (6)
C7	0.0397 (7)	0.0658 (9)	0.0499 (8)	0.0079 (6)	0.0147 (6)	0.0195 (7)
C8	0.0490 (8)	0.0517 (8)	0.0469 (7)	−0.0062 (6)	0.0107 (6)	−0.0010 (6)
C9	0.0407 (7)	0.0635 (9)	0.0411 (7)	0.0017 (6)	0.0039 (5)	0.0014 (6)
C10	0.0327 (5)	0.0359 (6)	0.0404 (6)	−0.0076 (5)	0.0059 (5)	0.0002 (5)
C11	0.0499 (8)	0.0397 (7)	0.0735 (10)	−0.0066 (6)	0.0131 (7)	−0.0083 (7)
C12	0.0713 (11)	0.0434 (8)	0.0759 (11)	−0.0228 (8)	0.0088 (9)	−0.0078 (7)
C13	0.0513 (8)	0.0660 (9)	0.0443 (7)	−0.0296 (7)	−0.0026 (6)	0.0081 (7)
C14	0.0316 (6)	0.0692 (10)	0.0534 (8)	−0.0112 (6)	0.0038 (5)	0.0085 (7)
C15	0.0320 (6)	0.0447 (7)	0.0500 (7)	−0.0045 (5)	0.0066 (5)	0.0028 (5)
C16	0.0732 (12)	0.1026 (16)	0.0695 (11)	−0.0573 (12)	−0.0065 (9)	0.0100 (11)

### 1-[N-(4-methylphenyl)-N-(methylsulfonyl)alanyl]piperidine-4-carboxylic acid (II) Geometric parameters (Å, º)

**Table:**

S1—O4	1.4309 (10)	C5—C8	1.508 (2)
S1—O5	1.4385 (10)	C5—C6	1.5252 (19)
S1—N1	1.6249 (10)	C5—H5	0.9800
S1—C17	1.7543 (15)	C6—C7	1.517 (2)
O1—C2	1.2300 (16)	C6—H6A	0.9700
O2—C8	1.2018 (19)	C6—H6B	0.9700
O3—C8	1.3269 (19)	C7—H7A	0.9700
O3—H3	0.90 (3)	C7—H7B	0.9700
N1—C10	1.4438 (15)	C9—H9A	0.9600
N1—C1	1.4834 (15)	C9—H9B	0.9600
N2—C2	1.3470 (17)	C9—H9C	0.9600
N2—C7	1.4677 (19)	C10—C15	1.3829 (18)
N2—C3	1.4688 (17)	C10—C11	1.3850 (19)
C1—C9	1.5238 (18)	C11—C12	1.390 (2)
C1—C2	1.5356 (17)	C11—H11	0.9300
C1—H1	0.9800	C12—C13	1.377 (3)
C17—H17A	0.9600	C12—H12	0.9300
C17—H17B	0.9600	C13—C14	1.382 (2)
C17—H17C	0.9600	C13—C16	1.513 (2)
C3—C4	1.5198 (19)	C14—C15	1.3882 (18)
C3—H3A	0.9700	C14—H14	0.9300
C3—H3B	0.9700	C15—H15	0.9300
C4—C5	1.531 (2)	C16—H16A	0.9600
C4—H4A	0.9700	C16—H16B	0.9600
C4—H4B	0.9700	C16—H16C	0.9600
			
O4—S1—O5	118.24 (6)	C7—C6—C5	110.50 (13)
O4—S1—N1	108.76 (6)	C7—C6—H6A	109.5
O5—S1—N1	105.74 (6)	C5—C6—H6A	109.5
O4—S1—C17	107.33 (7)	C7—C6—H6B	109.5
O5—S1—C17	107.40 (7)	C5—C6—H6B	109.5
N1—S1—C17	109.13 (7)	H6A—C6—H6B	108.1
C8—O3—H3	105.0 (16)	N2—C7—C6	110.97 (12)
C10—N1—C1	122.13 (10)	N2—C7—H7A	109.4
C10—N1—S1	118.32 (8)	C6—C7—H7A	109.4
C1—N1—S1	119.22 (8)	N2—C7—H7B	109.4
C2—N2—C7	126.82 (11)	C6—C7—H7B	109.4
C2—N2—C3	119.62 (12)	H7A—C7—H7B	108.0
C7—N2—C3	113.53 (11)	O2—C8—O3	122.04 (15)
N1—C1—C9	111.04 (10)	O2—C8—C5	125.76 (15)
N1—C1—C2	111.26 (10)	O3—C8—C5	112.20 (13)
C9—C1—C2	109.42 (11)	C1—C9—H9A	109.5
N1—C1—H1	108.3	C1—C9—H9B	109.5
C9—C1—H1	108.3	H9A—C9—H9B	109.5
C2—C1—H1	108.3	C1—C9—H9C	109.5
S1—C17—H17A	109.5	H9A—C9—H9C	109.5
S1—C17—H17B	109.5	H9B—C9—H9C	109.5
H17A—C17—H17B	109.5	C15—C10—C11	119.68 (12)
S1—C17—H17C	109.5	C15—C10—N1	119.04 (11)
H17A—C17—H17C	109.5	C11—C10—N1	121.26 (12)
H17B—C17—H17C	109.5	C10—C11—C12	119.43 (15)
O1—C2—N2	122.37 (12)	C10—C11—H11	120.3
O1—C2—C1	120.17 (11)	C12—C11—H11	120.3
N2—C2—C1	117.44 (11)	C13—C12—C11	121.70 (16)
N2—C3—C4	110.77 (12)	C13—C12—H12	119.1
N2—C3—H3A	109.5	C11—C12—H12	119.1
C4—C3—H3A	109.5	C12—C13—C14	118.04 (13)
N2—C3—H3B	109.5	C12—C13—C16	120.88 (18)
C4—C3—H3B	109.5	C14—C13—C16	121.08 (18)
H3A—C3—H3B	108.1	C13—C14—C15	121.38 (15)
C3—C4—C5	111.06 (12)	C13—C14—H14	119.3
C3—C4—H4A	109.4	C15—C14—H14	119.3
C5—C4—H4A	109.4	C10—C15—C14	119.76 (14)
C3—C4—H4B	109.4	C10—C15—H15	120.1
C5—C4—H4B	109.4	C14—C15—H15	120.1
H4A—C4—H4B	108.0	C13—C16—H16A	109.5
C8—C5—C6	111.72 (13)	C13—C16—H16B	109.5
C8—C5—C4	111.47 (12)	H16A—C16—H16B	109.5
C6—C5—C4	109.92 (11)	C13—C16—H16C	109.5
C8—C5—H5	107.9	H16A—C16—H16C	109.5
C6—C5—H5	107.9	H16B—C16—H16C	109.5
C4—C5—H5	107.9		
			
O4—S1—N1—C10	−39.35 (11)	C8—C5—C6—C7	−179.78 (12)
O5—S1—N1—C10	−167.31 (9)	C4—C5—C6—C7	−55.49 (15)
C17—S1—N1—C10	77.45 (11)	C2—N2—C7—C6	125.15 (15)
O4—S1—N1—C1	147.15 (9)	C3—N2—C7—C6	−56.90 (16)
O5—S1—N1—C1	19.20 (11)	C5—C6—C7—N2	56.02 (16)
C17—S1—N1—C1	−96.05 (11)	C6—C5—C8—O2	−1.4 (2)
C10—N1—C1—C9	20.64 (16)	C4—C5—C8—O2	−124.79 (17)
S1—N1—C1—C9	−166.13 (9)	C6—C5—C8—O3	179.68 (13)
C10—N1—C1—C2	−101.50 (13)	C4—C5—C8—O3	56.26 (17)
S1—N1—C1—C2	71.73 (12)	C1—N1—C10—C15	−106.70 (14)
C7—N2—C2—O1	179.61 (14)	S1—N1—C10—C15	80.01 (14)
C3—N2—C2—O1	1.8 (2)	C1—N1—C10—C11	71.61 (17)
C7—N2—C2—C1	1.4 (2)	S1—N1—C10—C11	−101.69 (14)
C3—N2—C2—C1	−176.43 (12)	C15—C10—C11—C12	0.5 (2)
N1—C1—C2—O1	28.24 (17)	N1—C10—C11—C12	−177.77 (14)
C9—C1—C2—O1	−94.83 (15)	C10—C11—C12—C13	−0.7 (3)
N1—C1—C2—N2	−153.51 (11)	C11—C12—C13—C14	0.0 (3)
C9—C1—C2—N2	83.42 (15)	C11—C12—C13—C16	179.38 (17)
C2—N2—C3—C4	−125.84 (14)	C12—C13—C14—C15	0.9 (2)
C7—N2—C3—C4	56.05 (17)	C16—C13—C14—C15	−178.47 (15)
N2—C3—C4—C5	−54.68 (15)	C11—C10—C15—C14	0.4 (2)
C3—C4—C5—C8	179.52 (11)	N1—C10—C15—C14	178.68 (12)
C3—C4—C5—C6	55.07 (15)	C13—C14—C15—C10	−1.1 (2)

### 1-[N-(4-methylphenyl)-N-(methylsulfonyl)alanyl]piperidine-4-carboxylic acid (II) Hydrogen-bond geometry (Å, º)

**Table:**

*D*—H···*A*	*D*—H	H···*A*	*D*···*A*	*D*—H···*A*
O3—H3···O5^i^	0.90 (3)	1.88 (3)	2.7463 (15)	161 (2)
C17—H17*A*···O1	0.96	2.48	3.144 (2)	127
C4—H4*B*···O2^i^	0.97	2.52	3.471 (2)	167
C11—H11···O1	0.93	2.56	3.2558 (19)	132

Symmetry code: (i) −*x*+2, −*y*+1, −*z*+1.
